# Estimativas de mortalidade por causas externas no Brasil, 2010-2019: metodologia de redistribuição de causas *garbage*

**DOI:** 10.1590/0102-311XPT056424

**Published:** 2024-11-22

**Authors:** Adauto Martins Soares, Renato Azeredo Teixeira, Ademar Barbosa Dantas, Juliana Bottoni de Souza, Marli de Mesquita Silva Montenegro, Ana Maria Nogales Vasconcelos, Maria de Fatima Marinho de Souza, Elisabeth Barboza França, Deborah Carvalho Malta

**Affiliations:** 1 Secretaria de Vigilância em Saúde e Ambiente, Ministério da Saúde, Brasília, Brasil.; 2 Programa de Pós-graduação em Saúde Coletiva, Universidade de Brasília, Brasília, Brasil.; 3 Indra Company, Belo Horizonte, Brasil.; 4 Programa de Pós-graduação em Saúde Pública, Universidade Federal de Minas Gerais, Belo Horizonte, Brasil.; 5 Instituto de Ciências Exatas, Universidade de Brasília, Brasília, Brasil.; 6 Vital Strategies Brazil, São Paulo, Brasil.; 7 Faculdade de Medicina, Universidade Federal de Minas Gerais, Belo Horizonte, Brasil.; 8 Escola de Enfermagem, Universidade Federal de Minas Gerais, Belo Horizonte, Brasil.

**Keywords:** Causas de Morte, Estatísticas Vitais, Causas Externas, Estudos Metodológicos, Confiabilidade dos Dados, Cause of Death, Vital Statistics, External Causes, Methodological Studies, Data Accuracy, Causas de Muerte, Estadísticas Vitales, Causas Externas, Estudios Metodológicos, Exactitud de los Datos

## Abstract

A qualidade insuficiente da causa básica de óbito no Sistema de Informações sobre Mortalidade (SIM) subenumera as violências, e se faz necessária a redistribuição das causas *garbage* (CG) em causas válidas na prevenção em saúde pública. Este estudo estimou a mortalidade de causas externas usando método de redistribuição de CG (GBD-Brasil), e comparou com dados do SIM e estimados do estudo GBD-IHME de 2010 a 2019 no Brasil e Unidades Federativas (UF). O algoritmo de redistribuição das CG do GBD-Brasil aplica etapas prévias do GBD-IHME com modificações, usando dois critérios: proporção das causas-alvo (válidas) ou reclassificação de causas investigadas. Os dados do SIM estão sem correção. Utiliza-se taxas padronizadas por método direto, regressão local na série temporal e razão das taxas GBD-Brasil e SIM como fator de correção para lesões de trânsito, quedas, suicídios e homicídios. O Brasil registrou 1,34 milhão de óbitos com causas externas válidas e 171.700 CG em 10 anos. A redistribuição de CG do GBD-Brasil aumentou em 12,2% as causas válidas, e a curva de tendência das taxas foi semelhante à encontrada com dados do SIM, mas divergem entre si e com o GBD-IHME em UFs do Norte e do Nordeste. As estimativas do GBD-Brasil mudaram o padrão das causas externas nas UFs, aplicando maiores correções em quedas nas UFs do Norte e do Nordeste e homicídios nas demais UFs. O método GBD-Brasil pode ser utilizado na análise de mortes violentas por buscar maior simplicidade metodológica, que garante tanto replicação por gestores públicos como consistência do dado estimado, considerando a composição local do dado no processo de redistribuição.

## Abstract (en)

Insufficient quality of the underlying cause of death in the Brazilian Mortality Information System (SIM), acronym in Portuguese underlists violence and it is necessary to redistribute garbage causes (GC) into valid causes in public health prevention. This study estimated mortality from external causes using the GC redistribution method (GBD-Brazil) and compared it with SIM and estimated data from the GBD-IHME study from 2010 to 2019 in Brazil and its states. The GBD-Brazil GC redistribution algorithm applies previous steps of the GBD-IHME with modifications, using two criteria: proportion of target causes (valid) or reclassification of investigated causes. The SIM data were not adjusted. Standardized rates by direct method, local regression in the time series, and the ratio of the GBD-Brazil and SIM rates are used as correction factors for traffic injuries, falls, suicides, and homicides. Brazil recorded 1.34 million deaths with valid external causes and 171,700 CG in ten years. The redistribution of GC from GBD-Brazil increased valid causes by 12.2%, and the trend curve of the rates was similar to that found with SIM data, but diverged from each other and from the GBD-IHME in states in the North and Northeast regions. The GBD-Brazil estimates changed the pattern of external causes in the states, applying greater corrections to falls in the states of the North and Northeast and homicides in the other states. The GBD-Brazil method can be used in the analysis of violent deaths because it seeks greater methodological simplicity, which guarantees both replication by public managers and consistency of the estimated data, considering the local composition of the data in the redistribution process.

## Abstract (es)

La calidad insuficiente de la causa de muerte en el Sistema de Información de Mortalidad brasileño (SIM) subestima la violencia, por lo cual es necesario redistribuir las causas *garbage* (CG) en causas válidas en la prevención de la salud pública. Este estudio estimó la mortalidad por causas externas por el método de redistribución de CG (GBD-Brasil) y la comparó con los datos de SIM y con los datos estimados del estudio GBD-IHME de 2010 a 2019 por el país (Brasil) y sus estados. El algoritmo de redistribución de CG del GBD-Brasil aplica los pasos anteriores de GBD-IHME con modificaciones, utilizando dos criterios: proporción de causas objetivo (válidas) o reclasificación de las causas investigadas. Los datos del SIM no están corregidos. Las tasas estandarizadas se utilizan método directo, regresión local en las series de tiempo y relación de tasas GBD-Brasil y SIM como factor de corrección para lesiones de tráfico, caídas, suicidios y homicidios. Brasil registró 1,34 millones de muertes por causas externas válidas y 171.700 CG en diez años. La redistribución de CG de GBD-Brasil aumentó las causas válidas en un 12,2%, y la curva de tendencia de las tasas fue similar a la encontrada con los datos SIM, pero divergieron entre sí y con el GBD-IHME en los estados del Norte y Nordeste. Las estimaciones del GBD-Brasil cambiaron el patrón de causas externas en los estados mediante la aplicación de mayores correcciones a las caídas en los estados del Norte y Nordeste y a los homicidios en los otros estados. El método GBD-Brasil se puede utilizar en el análisis de muertes violentas porque es una metodología más simple, lo que garantiza tanto la replicación por parte de los gestores públicos como la consistencia de los datos estimados, teniendo en cuenta la composición local de los datos en el proceso de redistribución.

## Introdução

O aperfeiçoamento das estatísticas vitais sobre mortalidade resultou em ampliação da base de dados coletada e redução das causas mal definidas no Brasil ^1^. Essas e outras causas *garbage* (CG), entretanto, representam um desafio na interpretação dos indicadores de violências e acidentes por provocar subenumeração das causas específicas de morte ^2^ ^,^ ^3^.

CG são um conjunto de códigos da Classificação Internacional de Doenças - 10ª Revisão (CID-10) que definem diagnósticos pouco específicos ou desconhecidos de causa básica de morte. A imprecisão das CG influencia o verdadeiro padrão de mortalidade e tem sido usada como indicador do nível de abrangência e especificidade dos dados por limitar sua utilidade para informar políticas de saúde pública ^4^. Essa falha no registro dos óbitos tem sido alvo de correção em estudos que objetivam extensas comparações internacionais ^5^ ^,^ ^6^.

O uso generalizado de CG na causa básica subenumera as causas específicas de mortes violentas. Por exemplo, traumatismo múltiplo como causa básica na declaração de óbito é CG, pois na verdade seria a lesão fatal a ser incluída como causa intermediária ou terminal decorrente de alguma circunstância que iniciou a cadeia de eventos que levou à morte, como acidentes, homicídio ou suicídio, verdadeiras causas básicas externas ^7^.

Em condições ideais, os dados de óbito teriam qualidade suficiente, e não seria necessário um método para redistribuição de CG ^8^. Como princípio geral, essas causas não deveriam exceder 10% das mortes em maiores de 65 anos e 5% nas idades inferiores ^9^. A confiabilidade dos dados permitiria a produção da evidência significativa para informar políticas públicas corretas de proteção à vida. Embora estatísticas de mortalidade sólidas e precisas não sejam por si só suficientes, a ausência delas deixaria espaço a especulações na tomada de decisões ^10^.

Entretanto, a questão das CG tem sido uma limitação compartilhada por diferentes países. Essas causas não especificadas ou implausíveis atingiram 29% dos óbitos na Noruega (entre 1996 e 2019) ^11^ e 25% nos Estados Unidos (entre 1980 e 2014) ^12^ e Coreia do Sul (entre 2010 e 2012) ^13^. Segundo o estudo *Carga Global da Doença* de 2010 (*Global Burden of Disease*; GBD-2010), entre 187 países, a CG foi mais baixa na Finlândia (5,5%) e mais alta no Sri Lanka (69,6%) ^5^. No último estudo GBD-2019, as CG de maior gravidade (classes 1 e 2) apresentaram valores substanciais na Argentina (27%), França e Uruguai (20%), Peru (19%), Japão e Brasil (17%), Estados Unidos (14%), México e Canadá (11%), Chile (10%), Colômbia (9%) e Cuba (8%) ^6^. Quantos às variações subnacionais, as menores foram registradas no Japão, Noruega e Reino Unido e, inversamente, as maiores foram na Rússia (5,1% a 27,7%) e no Brasil, variando de 8,5% no Espírito Santo à 29,5% na Bahia ^8^.

As lesões por causas externas são a principal razão de mortes prematuras no Brasil. Em 2017, os 158,7 mil registros de mortes violentas, considerados sensíveis a políticas públicas de promoção da saúde, revelam sua importância na agenda nacional de saúde pública. Parte desses registros, contudo, apresenta descrição inespecífica sobre a circunstância que levou ao óbito. De fato, mesmo após qualificação sistemática dos registros de mortalidade pela vigilância epidemiológica, a carga de *garbage* permaneceu expressiva, correspondendo a 14% (21,5 mil) das causas externas em 2017 ^2^ ^,^ ^14^. Como consequência, as CG de externas superam os suicídios no âmbito nacional, os homicídios no Estado de São Paulo, e os acidentes de transporte e suicídios em São Paulo e Rio de Janeiro ^15^. Estima-se ainda que 39% dos óbitos com intenção indeterminada são por homicídios ^3^. Um fator que dificulta a obtenção e a produção de dados de alta qualidade das causas externas é a emissão e a investigação das causas de morte fora do setor de saúde, envolvendo outras partes interessadas como médico-legista, polícia e sistema judicial ^16^ ^,^ ^17^.

Num cenário de incerteza da causa de mortalidade, além de medidas para o aperfeiçoamento do preenchimento mais completo das circunstâncias de causa de morte na declaração de óbito, outra dimensão a ser equacionada é o emprego de métodos confiáveis e adequados para a correção das causas externas. Diferentes estratégias de redistribuição das CG para estimar causas válidas são utilizadas com o propósito de compreender as causas básicas reais de mortalidade. Os métodos têm empregado modelos estatísticos, distribuição proporcional das causas definidas ou fatores de correção com base em dados empíricos. Assim, pretendem uma padronização da qualidade e da validade dos dados de mortalidade, possibilitando a comparabilidade geográfico-temporal das estatísticas de morte ^8^ ^,^ ^18^ ^,^ ^19^.

O GBD, estudo abrangente de estimativas epidemiológicas, aplica métodos para o tratamento dos dados de mortalidade, sendo a redistribuição das CG a etapa mais impactante ^8^, mas assume a desvantagem de ser complexo e de difícil reprodução ^18^. Com isso, é necessário investir em estratégias metodológicas mais acessíveis a formuladores de políticas em saúde para o uso oportuno da informação, mantendo as estimativas robustas e confiáveis. A fim de contribuir com o desenvolvimento de métodos acessíveis, que considerem a realidade local dos dados do Sistema de Informações sobre Mortalidade (SIM), este estudo objetiva estimar a mortalidade de causas externas por meio de uma proposta metodológica baseada em algoritmo de redistribuição de CG, e comparar com dados diretos do SIM e estimados do estudo GBD para o Brasil e Unidades Federativas (UFs) entre 2010 e 2019.

## Métodos

Foi feito um estudo descritivo-comparativo com o intuito de apresentar uma metodologia original e inovadora para gerar estimativas de causas externas de mortalidade corrigidas por algoritmo de redistribuição de CG em UFs brasileiras. Essas estimativas foram comparadas com dados do SIM, sem correção, e as estimativas do estudo GBD-IHME (do Instituto de Métricas e Avaliação em Saúde, Estados Unidos) entre 2010 e 2019.

O SIM do Ministério da Saúde ^20^ é a fonte primária dos óbitos utilizados neste estudo (GBD-Brasil/Universidade Federal de Minas Gerais [UFMG]). A comparação das estimativas geradas pelo estudo com os dados diretos avalia o impacto das correções nos dados oficiais de mortalidade do país. Por outro lado, visando uma referência comparativa com dados corrigidos, foram utilizadas as estimativas do GBD-IHME ^21^.

O estudo GBD-IHME aplica métodos complexos para o tratamento dos dados de mortalidade, previamente detalhados ^6^ ^,^ ^8^. Para o Brasil, o SIM também é sua fonte principal de dados. Nesse processo de correção do GBD, destacamos etapas importantes. A primeira é o mapeamento das causas básicas de morte de acordo com a lista GBD por idade (códigos do GBD-2019. Material Suplementar - Quadro S1; https://cadernos.ensp.fiocruz.br/static//arquivo/suppl-e00056424_5279.pdf). Em seguida, é feita uma avaliação das causas incoerentes (por exemplo, câncer de próstata em mulheres) e dados faltantes (sem idade ou sexo). A etapa que gera maior impacto nas estimativas é a redistribuição das CG, a qual considera o peso dessa ação para as suas respectivas causas-alvo, causas básicas válidas ou específicas finais. Por fim, são aplicadas técnicas para suavização dos dados levando em conta a variação estocástica ao longo dos anos, num processo chamado redução de ruído ^6^, e aplicados fatores de correção de sub-registro para as UFs. Todas as correções são feitas levando em consideração ano, sexo, idade e UFs do território brasileiro.

### Metodologia de redistribuição (GBD-Brasil)

O método de correção de óbitos do presente estudo executa procedimentos prévios da metodologia do GBD-IHME, nesta ordem: definição dos códigos da CID-10 mapeados e atribuídos para a lista de causas de morte do GBD (causas-alvo e *garbage*); redistribuição proporcional dos dados faltantes de idade e sexo, considerando local e ano; redistribuição das CG para causas-alvo; e aplicação dos fatores de correção do GBD-IHME para o sub-registro de óbitos por sexo e idade em cada UF do Brasil.

É importante destacar que na etapa de mapeamento das causas, segundo a lista GBD, a lista original foi modificada e adequada ao processo de redistribuição aplicado neste estudo. Uma vez que o estudo GBD define hierarquias de classes das CG e de níveis das causas específicas, definiu-se inicialmente o grau hierárquico a ser utilizado e se estabeleceu as causas específicas que seriam consideradas no estudo com uma avaliação para cada CG. Por exemplo, lesão de trânsito sem especificação do tipo de veículo ou modo de transporte da vítima são CG, entretanto, como foram calculadas estimativas para lesões de trânsito, essas CG foram pré-definida como lesão de trânsito, uma vez que são redistribuídas apenas dentro dos tipos de lesão de trânsito, subgrupos de detalhamento que não foram estimados no presente estudo.

### Mapeamento das causas externas

As causas externas definidas são consideradas alvo para a redistribuição de CG (CID-10) nos seguintes subgrupos: (1) lesões de trânsito (V01-V04.9, V06-V80.9, V82-V82.9, V87.0-V87.9, V89); (2) acidentes por quedas (W00-W19.9); (3) suicídios (X60-X64.9, X66-X84.9, Y87.0); (4) homicídios (X85-Y09.9, Y87.1); (5) outras lesões de transporte (V05-V05.9, V81-V81.9, V83-V86.9, V88.0-V88.9, V90-V99); e (6) outras causas externas (W200-W469, W490-W60.9, W64-W70.9, W730-W74.9, W770-W77.9, W810-W82.9, W850-W94.9, W97.9, W990-X06.9, X08-X39.9, X46.0-X48.9, X50-X54.9, X57-X58.9, Y350-Y849, Y880-Y883, Y890-Y891) e um grupo de códigos de causas naturais definidos previamente como outras causas externas (L550-L558, L563, L568, L580, L581).

Os demais códigos do capítulo XX de causas externas (CID-10: V010 a Y899), não mapeados nos subgrupos anteriores, são CG a serem redistribuídos.

Este estudo apresenta resultados para o total de causas externas-alvo e os quatro subgrupos de causa (lesões de trânsito, quedas, suicídios e homicídios). A lista completa de causas-alvo está disponível no Material Suplementar (Quadro S2; https://cadernos.ensp.fiocruz.br/static//arquivo/suppl-e00056424_5279.pdf).

### Procedimentos de redistribuição

**Figura 1:**
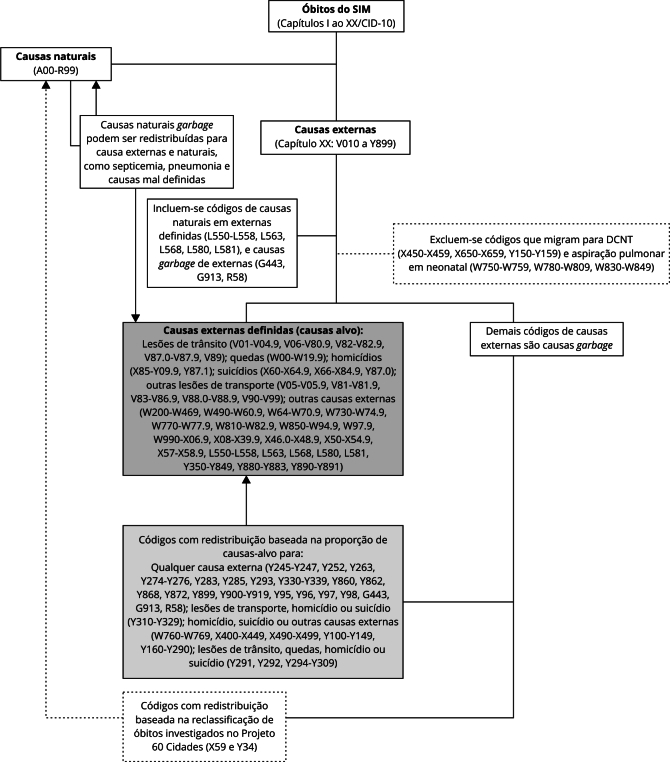
Algoritmo de redistribuição das causas externas *garbage* no método GBD-Brasil.

CID-10: Classificação Internacional de Doenças - 10ª Revisão; DCNT: doenças crônicas não transmissíveis; SIM: Sistema de Informações sobre Mortalidade.

Inicialmente, foram redistribuídos registros com dados faltantes de idade, sexo e ano. Após a análise das CG e respectivas causas específicas, a redistribuição das CG teve como criação dos pesos de redistribuição dois critérios principais: a proporção das causas-alvo em cada localidade, segundo sexo, idade e ano; ou a proporção de reclassificação das CG para causas-alvo considerando uma análise dos óbitos investigados pelo Projeto 60 Cidades ^2^. A escolha entre esses critérios teve como fatores o número de óbitos das CG e a diferença de resultado entre esses. Exposição acidental a fatores não especificados (X59) e eventos não especificados de intenção não determinada (Y34) foram redistribuídos pela reclassificação de óbitos investigados para causas-alvo de externas ou naturais (Material Suplementar - Quadro S3 e Tabela S1; https://cadernos.ensp.fiocruz.br/static//arquivo/suppl-e00056424_5279.pdf) ^22^. Destaca-se que as pneumonias, também redistribuídas pelo critério de óbitos investigados, mantiveram proporções consideráveis após o processo e, por esse motivo, foram mantidas como uma das causas-alvo ao fim da ação (Material Suplementar - Quadro S2; https://cadernos.ensp.fiocruz.br/static//arquivo/suppl-e00056424_5279.pdf). As demais causas externas *garbage* foram redistribuídas pela proporção das causas-alvo. Por sua vez, as causas externas-alvo ainda receberam óbitos redistribuídos de algumas causas naturais *garbage*, i.e. septicemias, pneumonias e causas mal definidas. Dados empíricos de reclassificação das CG confirmam esse processo de migração entre causas naturais e externas ^2^ ^,^ ^18^. O algoritmo de redistribuição das causas externas *garbage* no método GBD-Brasil é representado graficamente na Figura 1, e as rotinas de redistribuição das CG para os dados do SIM estão disponíveis em linguagem em R no link: https://github.com/saeufmg/gc_redistribution_ufmg.

O Projeto 60 Cidades foi uma intervenção brasileira para melhorar o diagnóstico da causa básica de óbito registrada no SIM em 2017. Entre as ações, a CG de externas foi investigada a partir de diferentes fontes de informação, incluindo institutos de medicina legal e hospitais, utilizando um formulário padrão de investigação. Os 60 municípios registraram cerca da metade dessas causas no país, das quais 65% foram reclassificadas após investigação. A base de dados de óbito do SIM das localidades com a informação das causas básicas antes e após a investigação serviu de parâmetro para a reclassificação e a obtenção de valores percentuais de mudança de uma CG de externa para uma causa válida e suficientemente específica para informar a circunstância da morte de causa externa ^2^. Então, as frações percentuais de reclassificação para o conjunto desses dados foram empregadas neste estudo, conforme valores apresentados no Material Suplementar (Quadro S3; https://cadernos.ensp.fiocruz.br/static//arquivo/suppl-e00056424_5279.pdf).

### Dados diretos do SIM

O cálculo do total de causas externas do SIM considera as causas externas específicas do SIM (lesões de trânsito, quedas, suicídios e homicídios), conforme a definição anterior de causas-alvo, e as causas externas *garbage*, que são redistribuídas apenas entre as causas externas. Portanto, a organização dos dados diretos de causas externas do SIM (capítulo XX da CID-10: V010-Y899) segue as etapas descritas a seguir:

Etapa 1: Foram incluídas na base de dados de causas externas um grupo de causas naturais considerado causas externas definidas (L550-L558, L563, L568, L580, L581) e outro listado como CG de causas externas (G443, G913, R58);

Etapa 2: Foram excluídos da base de dados de causas externas um grupo de causas considerados doenças crônicas não transmissíveis (X450-X459, X650-X659, Y150-Y159) e outro como aspiração pulmonar em neonatal (W750-W759, W780-W809, W830-W849); e

Etapa 3: identificadas e excluídas as CG externas que podem ser redistribuídas para causas externas ou causas naturais, conforme resultados do Projeto 60 Cidades: exposição acidental a fatores não especificados (X59); e eventos não especificados de intenção não determinada (Y34) ^22^.

### Análise de dados

As taxas padronizadas de mortalidade por 100 mil habitantes, usando método direto, são utilizadas para comparação entre estimativas dos métodos GBD-Brasil e GBD-IHME e dados diretos do SIM em causas externas e seus quatro subgrupos (lesões de trânsito, quedas, suicídios e homicídios). A população utilizada nos três métodos está disponível no *site* do Ministério da Saúde, tendo a do ano 2010 como população padrão ^23^. Inicialmente, apresenta-se a série temporal das taxas de mortalidade de 2010 a 2019 no Brasil, aplicando o método de *loess* (regressão local), e por UFs, bem como fatores de correção dos dados diretos do SIM, usando a razão de taxas: r = GBD-Brasil / Dados do SIM. Um ranking das taxas exibe mudanças no perfil de mortalidade por UF com o método GBD-Brasil em relação aos dados diretos do SIM em 2019.

### Aspectos éticos

Esta pesquisa obedece à *Resolução nº 466/2012*, do Conselho Nacional de Saúde. A pesquisa foi aprovada pelo Comitê de Ética em Pesquisa envolvendo Seres Humanos da UFMG (parecer nº 3.258.076).

## Resultados

O Brasil registrou, de 2010 a 2019, uma carga de mortalidade inicial por causas externas de 1,34 milhão de óbitos com causas suficientemente especificadas e 171.700 CG. Entre as CG, 46,4% foram por exposição acidental a fatores não especificados (X59) ou eventos não especificados de intenção não determinada (Y34). A redistribuição de CG com o método GBD-Brasil aumentou as causas externas especificadas para 1,50 milhão de óbitos em 12,2% no período. Em etapa posterior, a correção de sub-registro elevou em mais 6% a carga nacional de mortalidade de causas externas para 1,59 milhão.

**Figura 2:**
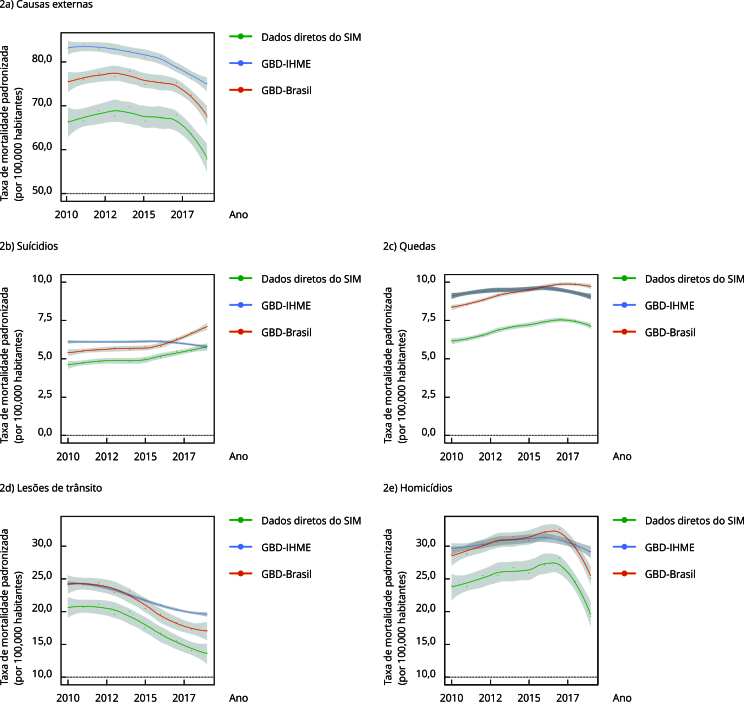
Taxas de mortalidade padronizadas por tipo de causas externas, segundo dados diretos do Sistema de Informações sobre Mortalidade (SIM), métodos de correção GBD-IHME e GBD-Brasil. Brasil, 2010 a 2019.

As taxas estimadas do GBD-Brasil evoluíram com traçado semelhante às curvas de tendência com dados diretos do SIM de 2010 a 2019, diferentemente do GBD-IHME, que tem estimativas temporalmente suavizadas. Os três métodos apontaram tendência de decréscimo das taxas de mortalidade por causas externas e lesões de trânsito para o âmbito nacional. No entanto, taxas de suicídios e mortes acidentais por quedas apresentaram tendência de aumento no GBD-Brasil (32,9%; 15,7%) e nos dados do SIM (26,3%; 14,6%), e os homicídios apresentaram redução (-12,6%; -19,6%), enquanto o GBD-IHME indicou estabilidade (Figura 2).

Fonte: Sistema de Informações sobre Mortalidade ^20^; Instituto de Métricas e Avaliação em Saúde ^21^.

Os métodos aplicados convergem com a tendência negativa das taxas de mortalidade de causas externas em todos as UFs do Sudeste, do Sul e do Centro-oeste, embora com diferenças marcantes de magnitude entre as unidades geográficas, sobretudo no Distrito Federal e no Mato Grosso. No Norte e no Nordeste, os métodos convergem com tendência também negativa no Pará, em Rondônia, no Alagoas e na Paraíba, e positiva no Acre, em Roraima e no Piauí (Tabela 1).

**Tabela 1:** Taxa padronizada de mortalidade por causas externas e variação percentual, segundo dados diretos do Sistema de Informações sobre Mortalidade (SIM), métodos de correção GBD-IHME e GBD-Brasil, e Unidades Federativas (UF). Brasil, 2010 e 2019.

Região/UFs	Dados diretos do SIM		Taxa corrigida				Variação percentual (2010-2019)		
			GBD-IHME		GBD-Brasil				
	2010	2019	2010	2019	2010	2019	SIM	GBD-IHME	GBD-Brasil
Brasil	66,5	57,8	83,1	75,2	75,6	67,4	-13,1	-9,6	-10,9
Norte									
Acre	65,0	68,2	75,3	79,0	69,2	73,2	4,9	4,9	5,8
Amapá	73,8	72,1	75,5	78,0	77,4	77,6	-2,3	3,3	0,3
Amazonas	66,0	71,1	77,1	70,5	71,3	74,0	7,8	-8,6	3,7
Pará	76,5	71,0	93,3	83,7	84,1	77,0	-7,2	-10,2	-8,5
Rondônia	97,1	70,1	103,0	94,7	104,7	89,2	-27,8	-8,0	-14,8
Roraima	82,5	100,6	84,2	88,2	91,1	106,9	22,0	4,7	17,3
Tocantins	84,3	82,5	86,1	82,8	90,9	95,7	-2,1	-3,9	5,3
Nordeste									
Alagoas	103,8	68,3	129,4	102,1	119,5	84,4	-34,2	-21,1	-29,4
Bahia	74,4	75,7	94,1	88,0	89,0	87,7	1,6	-6,4	-1,5
Ceará	73,4	61,5	98,3	104,3	94,0	77,0	-16,2	6,1	-18,0
Maranhão	58,0	61,6	87,5	87,8	74,8	79,3	6,2	0,4	6,1
Paraíba	70,3	60,3	92,4	82,9	80,3	68,3	-14,1	-10,3	-14,9
Pernambuco	75,4	71,4	100,9	100,6	91,0	92,0	-5,3	-0,3	1,1
Piauí	59,9	62,0	68,4	73,2	65,3	69,8	3,4	7,0	7,0
Rio Grande do Norte	59,5	65,9	80,7	86,6	76,8	71,7	10,8	7,3	-6,7
Sergipe	82,0	79,2	91,6	100,9	86,1	88,2	-3,4	10,2	2,4
Sudeste									
Espírito Santo	101,6	69,9	115,0	95,1	112,3	90,0	-31,2	-17,3	-19,8
Minas Gerais	56,6	49,9	77,2	68,3	66,1	60,3	-11,8	-11,5	-8,7
Rio de Janeiro	68,4	50,6	94,0	81,5	79,8	69,5	-26,1	-13,4	-12,9
São Paulo	50,1	38,7	62,8	52,4	55,7	43,7	-22,7	-16,6	-21,5
Sul									
Paraná	84,4	66,4	93,2	76,3	88,4	71,7	-21,4	-18,2	-18,9
Rio Grande do Sul	59,8	57,3	69,0	65,6	63,1	60,6	-4,2	-4,9	-4,0
Santa Catarina	60,6	54,3	71,4	60,2	65,6	58,3	-10,3	-15,6	-11,1
Centro-oeste									
Distrito Federal	71,0	47,0	73,7	59,2	76,4	54,2	-33,7	-19,6	-29,0
Goiás	82,1	77,3	95,2	93,0	90,0	87,6	-5,8	-2,3	-2,6
Mato Grosso	92,2	76,4	99,7	89,8	96,5	81,4	-17,2	-10,0	-15,6
Mato Grosso do Sul	81,9	63,2	87,3	72,3	84,2	66,5	-22,8	-17,1	-21,1

Fonte: Sistema de Informações sobre Mortalidade ^20^; Instituto de Métricas e Avaliação em Saúde ^21^.

Ao comparar as taxas corrigidas em relação às taxas com os dados diretos, observou-se que a correção do GBD-IHME teve maior impacto do que a metodologia do GBD-Brasil no âmbito nacional, exceto no último triênio para suicídios, mortes por quedas e homicídios. Em ambos os métodos, os fatores de correção das taxas de mortalidade de causas externas variaram com redução até 2018, voltando a aumentar em 2019 (Figura 2 e Tabela 2). Analisando o método GBD-Brasil, observou-se uma maior correção nas taxas para o último ano em todas as causas. Contudo, o fator de correção foi menor em 2019 em algumas UFs, particularmente na Paraíba e no Rio Grande do Norte, para todas as causas. Acidentes por quedas apresentaram mais UFs com correção menor em 2019 (14 de 27), enquanto 23 de 27 UFs exibiram correção maior para homicídios (Tabela 2).

**Tabela 2:** Fator de correção das taxas padronizadas de mortalidade com dados diretos do Sistema de Informações sobre Mortalidade (SIM) usando o método GBD-Brasil, segundo causas externas, Brasil e Unidades Federativas (UF), 2010 e 2019.

Região/UFs	Causas externas		Lesões de trânsito		Quedas		Homicídios		Suicídios	
	2010	2019	2010	2019	2010	2019	2010	2019	2010	2019
Brasil	1,14	1,17	1,17	1,25	1,35	1,37	1,20	1,30	1,17	1,23
Norte										
Acre	1,06	1,07	1,06	1,08	1,12	1,44	1,10	1,07	1,10	1,08
Amapá	1,05	1,08	1,05	1,14	1,75	1,24	1,06	1,07	1,09	1,07
Amazonas	1,08	1,04	1,09	1,06	1,20	1,21	1,10	1,09	1,09	1,08
Pará	1,10	1,08	1,12	1,10	1,37	1,30	1,10	1,12	1,13	1,15
Rondônia	1,08	1,27	1,10	1,27	1,18	1,35	1,11	1,38	1,10	1,30
Roraima	1,10	1,06	1,05	1,11	1,89	1,18	1,16	1,18	1,10	1,13
Tocantins	1,08	1,16	1,08	1,18	1,16	1,28	1,10	1,19	1,12	1,21
Nordeste										
Alagoas	1,15	1,24	1,17	1,27	1,30	1,29	1,13	1,26	1,17	1,26
Bahia	1,20	1,16	1,31	1,28	1,78	1,70	1,23	1,25	1,38	1,35
Ceará	1,28	1,25	1,30	1,33	1,79	1,68	1,27	1,34	1,24	1,28
Maranhão	1,29	1,29	1,31	1,33	1,90	1,76	1,23	1,24	1,31	1,29
Paraíba	1,14	1,13	1,15	1,14	1,42	1,22	1,15	1,17	1,17	1,15
Pernambuco	1,21	1,29	1,27	1,44	1,69	1,50	1,25	1,40	1,29	1,40
Piauí	1,09	1,13	1,07	1,09	1,30	1,42	1,13	1,17	1,07	1,09
Rio Grande do Norte	1,29	1,09	1,35	1,13	1,84	1,50	1,38	1,11	1,27	1,16
Sergipe	1,05	1,11	1,08	1,19	1,15	1,29	1,10	1,20	1,12	1,23
Sudeste										
Espírito Santo	1,11	1,29	1,12	1,38	1,17	1,32	1,14	1,45	1,14	1,37
Minas Gerais	1,17	1,21	1,18	1,26	1,36	1,30	1,25	1,40	1,20	1,21
Rio de Janeiro	1,17	1,38	1,24	1,93	1,42	2,32	1,25	1,72	1,23	1,63
São Paulo	1,11	1,13	1,17	1,30	1,34	1,38	1,27	1,62	1,19	1,33
Sul										
Paraná	1,05	1,08	1,06	1,13	1,13	1,12	1,10	1,26	1,06	1,18
Rio Grande do Sul	1,05	1,06	1,08	1,09	1,28	1,13	1,16	1,11	1,11	1,08
Santa Catarina	1,08	1,07	1,10	1,10	1,19	1,13	1,12	1,17	1,07	1,12
Centro-oeste										
Distrito Federal	1,08	1,15	1,09	1,21	1,10	1,13	1,16	1,28	1,09	1,21
Goiás	1,10	1,13	1,09	1,15	1,18	1,16	1,14	1,19	1,10	1,14
Mato Grosso	1,05	1,07	1,05	1,09	1,12	1,12	1,11	1,14	1,14	1,11
Mato Grosso do Sul	1,03	1,05	1,05	1,09	1,18	1,28	1,09	1,16	1,10	1,14

Fonte: Sistema de Informações sobre Mortalidade ^20^; Instituto de Métricas e Avaliação em Saúde ^21^.

O GBD-Brasil estimou maior correção da taxa de mortalidade acidental por quedas no Brasil e nas UFs do Norte e do Nordeste em 2019, exceto Rondônia, que foi homicídio (1,38). Nas UFs das demais regiões, o homicídio obteve maior correção, exceto Rio de Janeiro (2,32), Mato Grosso do Sul (1,28) e Rio Grande do Sul (1,13), que foram mortes acidentais por quedas (Tabela 2). Entre as UFs, as maiores correções em cada tipo de causa foram: (1) mortes de lesões de trânsito e suicídios no Rio de Janeiro, em Pernambuco e no Espírito Santo; (2) mortes acidentais por quedas no Rio de Janeiro, no Maranhão e na Bahia; (3) e homicídios no Rio de Janeiro, em São Paulo e no Espírito Santo.

**Figura 3:**
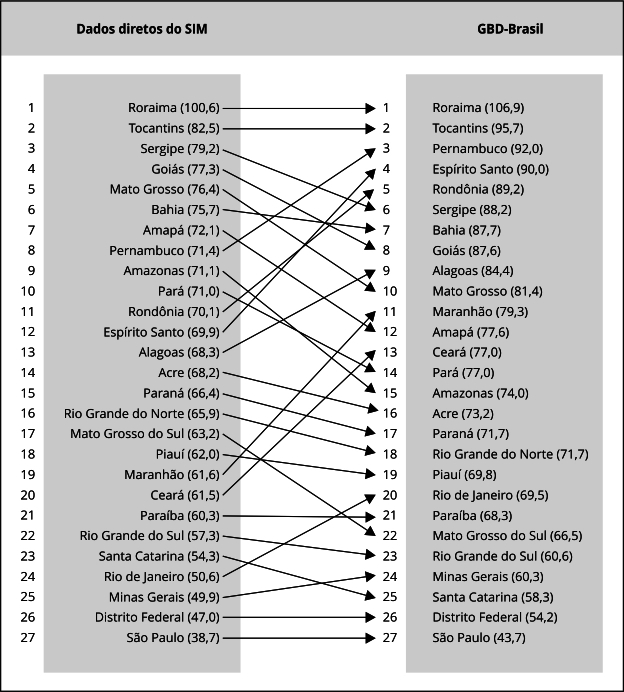
Classificação das taxas padronizadas de causas externas com dados diretos do Sistema de Informações sobre Mortalidade (SIM) e o método GBD-Brasil, segundo Unidades Federativas, Brasil, 2019.

A correção dos dados diretos do SIM com o método GBD-Brasil mudou a magnitude das taxas de mortalidade por causas externas e a posição de classificação de risco em 22 das 27 UFs em 2019 (Figura 3). Além da Paraíba, as UFs com as maiores taxas (Roraima e Tocantins) e menores (Distrito Federal e São Paulo) mantiveram suas posições. As UFs que mais subiram de posição foram Espírito Santo (12º para 4º), Maranhão (19º para 11º) e Ceará (20º para 13º). Enquanto os que desceram mais posições foram Amazonas (9º para 15º), Amapá (7º para 12º), Mato Grosso (5º para 10º) e Mato Grosso do Sul (17º para 22º). Esse padrão de mudança se reproduz nos subgrupos de causas externas (Material Suplementar - Quadros S4, S5, S6 e S7; https://cadernos.ensp.fiocruz.br/static//arquivo/suppl-e00056424_5279.pdf). As UFs que mais subiram de posição foram em lesões de trânsito (Espírito Santo, Rio de Janeiro e Pernambuco), homicídios (Pernambuco, Alagoas e Rio de Janeiro), mortes acidentais por quedas (Rio de Janeiro, Pernambuco, Ceará e Maranhão) e suicídios (Espírito Santo, Rondônia, Minas Gerais e São Paulo).

Fonte: Sistema de Informações sobre Mortalidade ^20^; Instituto de Métricas e Avaliação em Saúde ^21^.

## Discussão

Os métodos GBD-Brasil e GBD-IHME redistribuíram quantidade substancial de causas externas *garbage* derivada dos dados diretos do SIM. No âmbito nacional, a tendência das taxas de mortalidade do GBD-Brasil e dados diretos do SIM convergiram com curvas semelhantes, embora as magnitudes sejam distintas. No nível subnacional, os três métodos divergiram entre si nos resultados de tendências das taxas em UFs do Norte e Nordeste. O processo de correção do GBD-Brasil afeta o número de óbitos e as taxas de mortalidade nas UFs atribuídos para cada tipo de causa externa do estudo, resultando em alteração na classificação de risco entre esses locais. O método aplica correções maiores em acidentes por quedas nas UFs do Norte e Nordeste, por sua vez, corrigindo os homicídios em maior magnitude nas UFs das demais regiões.

Os resultados do estudo mostraram que as CG representam uma grande parcela das causas de morte no Brasil. Embora exista redução gradual dos fatores de correção do GBD-Brasil e GBD-IHME, ocorreu uma inflexão em 2019, indicando piora na qualidade do registro das causas externas de morte nesse ano. Ao mesmo tempo que apontaram excesso de óbitos notificados com CG no Brasil, achados indicaram uma tendência de redução que não persistiu após 2018 ^24^. Importante destacar que o ano de 2019 das estimativas do IHME utilizaram dados do SIM até 2018, sendo que os dados de 2019 ainda eram preliminares. Para esse ano, foram utilizados modelos do IHME para gerar as estimativas.

A recente piora na qualidade dos dados de causas externas foi particularmente relevante no ano de 2019. Após cair por um período de mais de 15 anos, eventos de intenção indeterminada voltaram a subir em 2019. A situação foi mais grave sobretudo no Estado do Rio de Janeiro ^25^. Esses dados corroboram nossos resultados, que indicaram fatores de correção maiores para a referida UF, independentemente do tipo de causa externa. O aumento dos *garbage* de causas externas no SIM prejudica ainda mais comparações com anos anteriores e entre áreas geográficas, além de exigir da vigilância epidemiológica municipal esforço adicional de recaptura de dados sobre as circunstâncias das causas externas ^15^.

Por sua vez, o declínio das CG até 2018 é explicado por uma carga decrescente dos *garbage* de maior gravidade entre 2006-2016, principalmente devido a redução das causas mal definidas de óbitos em UFs menos desenvolvidas do Norte e Nordeste do Brasil, mitigando desigualdades regionais na qualidade do dado ^26^. Localidades com índices sociodemográficos desfavorecidos tendem a exibir excesso de CG menos específicas (classes 1 e 2) ^8^.

As estimativas do GBD-Brasil acompanharam mudanças na notificação dos dados de causas externas no SIM ao longo da série em análise, diferente do que ocorre com as estimativas do GBD-IHME, que adota a suavização matemática da tendência dos dados. Apesar do GBD-Brasil reproduzir as curvas das taxas com dados diretos do SIM no nível nacional, as tendências divergiram em algumas UFs do Norte e Nordeste. Esse padrão subnacional indica que UFs dessas regiões seriam mais afetados pela qualidade do registro das causas externas. De modo semelhante, a correção de homicídios não alterou a trajetória dos dados, acompanhando a linha de tendência dos óbitos registrados no SIM, embora altere a magnitude das variações das taxas nos períodos em análise, conforme também verificamos. Como consequência das mudanças de magnitude, observam-se trocas de posição da classificação de risco entre as UFs ^3^.

Com o enfoque abrangente de redistribuição das CG a partir de adaptação da metodologia do estudo GBD, a correção das taxas de causas externas do método GBD-Brasil resulta em mudanças de posição na classificação de risco por mortes violentas em comparação aos dados diretos do SIM nas UFs. Como ilustração do efeito da redistribuição das CG em dados nacionais, as causas líderes de mortalidade mudaram de posição em países com distinta fase de desenvolvimento, como o Brasil, México e Uruguai, e os Estados Unidos, Japão e França, indicando a necessidade de múltiplos países considerarem a contabilização das CG nas estatísticas de mortalidade ^5^ ^,^ ^27^. O mesmo foi verificado em estudos específicos com dados da Coreia do Sul ^10^ e Brasil ^20^. Quando a CG é frequente, as correções também são necessárias ao devido ajuste da tendência das causas de morte, conforme o ajuste obtido da tendência subnacional nos Estados Unidos ^12^.

Por ainda não exibirem padrões desejáveis de qualidade e confiança nos dados de causas externas ^9^ ^,^ ^10^, faz-se duplamente necessária a qualificação dos registros ^22^ ^,^ ^26^ e o uso de estimativas de mortalidade úteis para políticas públicas ^18^. O Brasil deve ampliar iniciativas de aprimoramento sistemático do registro das causas de mortes violentas que incluam trabalho intersetorial da saúde e segurança pública ^17^. Simultaneamente, deve avançar em métodos de correção de óbitos que incorporem cada vez mais dados empíricos ^2^ ^,^ ^8^ ^,^ ^19^ ^,^ ^22^ ^,^ ^28^, e sejam acessíveis aos gestores da saúde, como se propõe o método apresentado neste estudo.

Correções das informações sobre causas de morte empregam recursos estatísticos e empíricos ^22^. Em método matemático de previsão informada da causa básica de morte a partir de informações sobre causas contribuintes e características demográficas do falecido, o resultado da redistribuição foi diferente daquele que considera apenas as proporções ao nível da população ^29^. De modo semelhante, o método de redistribuição das causas externas indeterminadas identificou características associadas a cada incidente violento para predizer a intenção do evento ^28^. Por sua vez, um estudo ^19^ encontrou resultados distintos de redistribuição de CG ao aplicar dois métodos empíricos, o relacionamento de registros de internação e mortalidade, e o uso de informações das causas múltiplas de óbito que também considera causas contribuintes e características do indivíduo. A aplicação de métodos variados para a redistribuição de CG no GBD-2019, indo da análise de dados de causas múltiplas à redistribuição proporcional, representou uma melhoria geral no empirismo em comparação com a dependência anterior do conhecimento *a priori* ^8^.

O método proposto no presente artigo leva em consideração as realidades nacionais. Considerando a redistribuição proporcional, cada localidade apresenta sua distribuição proporcional por causas, em especial ao serem considerados os municípios, a idade e o sexo. Isso faz com que a heterogeneidade brasileira seja considerada no processo de redistribuição. Ainda levando em conta uma realidade nacional, ao levar em conta o uso de pesos baseados nas investigações de óbitos por CG do Projeto 60 Cidades, usa-se dados empíricos do país na avaliação das investigações para as causas por exposição acidental a fatores não especificados, eventos não especificados de intenção não determinada e pneumonias ^2^ ^,^ ^22^. O Brasil é um dos poucos países que fazem investigações de óbitos de forma rotineira, sendo anualmente mais de 170 mil investigados desde 2010. A análise desses dados pode gerar novos pesos baseados em dados empíricos nacionais, visando expandir e atualizar os métodos de redistribuição que vêm sendo utilizados ^30^.

As limitações deste estudo se relacionam ao emprego restrito de dados empíricos para determinar a redistribuição de CG e gerar atribuições de causas de morte mais plausíveis. Dois códigos de causas externas *garbage* foram redistribuídos com base nos dados de reclassificação por investigação de campo, os demais por proporção da causa externa definida. Comumente, a reclassificação das CG para uma causa básica mais precisa de óbito num processo de recuperação de dados de campo não segue o mesmo padrão de distribuição das causas definidas registradas no SIM ^2^ ^,^ ^18^ ^,^ ^19^ ^,^ ^22^. Uma aplicação mais ampla dos dados da investigação pode ser um recurso a ser testado no aperfeiçoamento do método GBD-Brasil. Por fim, para contornar eventuais dificuldades de acesso e uso do algoritmo por usuários finais, um repositório foi criado para compartilhar as informações metodológicas e as técnicas do processo de redistribuição de causas garbage apresentadas neste estudo, bem como receber contribuições ao seu aprimoramento.

Com dados confiáveis, os esforços para planejar o desenvolvimento e o bem-estar da população podem ser fundamentados na realidade e, portanto, tornam-se mais assertivos. Enquanto persistirem deficiências de qualidade dos dados, a implementação de metodologias de correção se faz necessária. O algoritmo apresentado neste estudo se coloca como uma opção de método a ser utilizado na análise de estatísticas de violências e acidentes, em particular ao se buscar uma maior simplicidade metodológica possível, que garanta ao mesmo tempo a sua replicação por gestores públicos e a consistência do dado de mortalidade estimado.
