# Supplementary material for: Estimates of mortality from external causes in Brazil, 2010-2019: methodology for redistribution of garbage causes
Source: Cad Saude Publica. 2024 Nov 22;40(10):e00056424. [Article in Portuguese] doi: 10.1590/0102-311XPT056424 (PMC11654112; doi:10.1590/0102-311XPT056424)
Supplement: Supplementary file 1 [file 1678-4464-csp-40-10-PT056424-s.pdf]

MATERIAL SUPLEMENTAR

Quadro S1: Lista dos códigos da CID-10 mapeados para a lista de causas de GBD por causa de morte.

List of International Classification of Diseases (ICD) codes mapped to the Global Burden of Disease cause list for causes of death	
**Cause**	**ICD10**
Injuries	L55-L55.9, L56.3, L56.8-L56.9, L58-L58.9, N30.4, U00-U03, V00-V86.9, V87.2-V87.3, V88.2-V88.3, V90-V98.8, W00-W46.2, W49-W62.9, W64-W70.9, W73-W75.9, W77-W81.9, W83-W94.9, W97.9, W99-X06.9, X08-X39.9, X47-X48.9, X50-X54.9, X57-X58.9, X60-X64.9, X66-X83.9, X85-Y08.9, Y35-Y84.9, Y87.0-Y87.1, Y88-Y88.3, Y89.0-Y89.1
Transport injuries	V00-V86.9, V87.2-V87.3, V88.2-V88.3, V90-V98.8
Road injuries	V01-V04.9, V06-V80.9, V82-V82.9, V87.2-V87.3
Pedestrian road injuries	V01-V04.9, V06-V09.9
Cyclist road injuries	V10-V19.9
Motorcyclist road injuries	V20-V29.9
Motor vehicle road injuries	V30-V79.9, V87.2-V87.3
Other road injuries	V80-V80.9, V82-V82.9
Other transport injuries	V00-V00.8, V05-V05.9, V81-V81.9, V83-V86.9, V88.2-V88.3, V90-V98.8
Unintentional injuries	L55-L55.9, L56.3, L56.8-L56.9, L58-L58.9, N30.4, W00-W46.2, W49-W62.9, W64-W70.9, W73-W75.9, W77-W81.9, W83-W94.9, W97.9, W99-X06.9, X08-X39.9, X47-X48.9, X50-X54.9, X57-X58.9, Y40-Y84.9, Y88-Y88.3
Falls	W00-W19.9
Drowning	W65-W70.9, W73-W74.9
Fire, heat, and hot substances	X00-X06.9, X08-X19.9
Poisonings	X47-X48.9
Poisoning by carbon monoxide	X47-X47.9
Poisoning by other means	X48-X48.9
Exposure to mechanical forces	W20-W38.9, W40-W43.9, W45.0-W45.2, W46-W46.2, W49-W52
Unintentional firearm injuries	W32-W34.9
Other exposure to mechanical forces	W20-W31.9, W35-W38.9, W40-W43.9, W45.0-W45.2, W46-W46.2, W49-W52
Adverse effects of medical treatment	N30.4, Y40-Y84.9, Y88-Y88.3
Animal contact	W52.0-W62.9, W64-W64.9, X20-X29.9
Venomous animal contact	X20-X29.9
Non-venomous animal contact	W52.0-W62.9, W64-W64.9
Foreign body	W44-W45, W45.3-W45.9, W75-W75.9, W78-W80.9, W83-W84.9
Pulmonary aspiration and foreign body in airway	W75-W75.9, W78-W80.9, W83-W84.9
Foreign body in other body part	W44-W45, W45.3-W45.9
Environmental heat and cold exposure	L55-L55.9, L56.3, L56.8-L56.9, L58-L58.9, W88-W94.9, W97.9, W99-W99.9, X30-X32.9, X39-X39.9
Exposure to forces of nature	X33-X38.9
Still Born	P95-P95.9
Other unintentional injuries	W39-W39.9, W77-W77.9, W81-W81.9, W85-W87.9, X50-X54.9, X57-X58.9
Self-harm and interpersonal violence	U00-U03, X60-X64.9, X66-X83.9, X85-Y08.9, Y35-Y38.9, Y87.0-Y87.1, Y89.0-Y89.1
Self-harm	X60-X64.9, X66-X83.9, Y87.0
Self-harm by firearm	X72-X74.9
Self-harm by other specified means	X60-X64.9, X66-X71.9, X75-X83.9, Y87.0
Interpersonal violence	X85-Y08.9, Y87.1
Physical violence by firearm	X93-X95.9
Physical violence by sharp object	X99-X99.9
Physical violence by other means	X85-X92.9, X96-X98.9, Y00-Y04.9, Y06-Y08.9, Y87.1
Conflict and terrorism	U00-U03, Y36-Y38.9, Y89.1
Police conflict and executions	Y35-Y35.9, Y89.0
Garbage Code (GBD Level 1)	A40-A41.9, A48.0, A48.3, A49.0-A49.1, A59-A59.9, A71-A71.9, A74.0, B07-B07.9, B30-B30.9, B35-B36.9, B85-B85.4, B87-B88.9, B94.0, D50-D50.0, D50.9, D62-D63.0, D63.8-D64, D64.1-D65.9, D68, D69.9, E15, E16, E50-E50.9, E64.1, E85.3-E87.6, E87.8-E87.9, F06.2-F06.4, F07.2, F09-F09.9, F19-F23.9, F25-F49, F51-F99.0, G06-G08.0, G32-G32.8, G43-G44.2, G44.4-G44.8, G47-G47.2, G47.4-G47.9, G50-G60.9, G62-G62.0, G62.2-G65.2, G80-G83.9, G89-G89.4, G91-G91.2, G91.4-G93, G93.1-G93.2, G93.4-G93.6, G94.0-G94.8, G99-H05, H05.2-H69.9, H71-H99, I26-I26.9, I31.2-I31.4, I46-I46.9, I50.0-I50.4, I76, I95-I95.1, I95.8-I95.9, J69-J69.9, J80-J80.9, J81.0, J85-J85.3, J86-J86.9, J93-J93.1, J93.8-J93.9, J94.2, J96-J96.9, J98.1-J98.3, K00-K19, K30, K65-K66.1, K66.9, K68.1-K68.9, K71-K71.6, K71.8-K72.9, K75.0, L20-L30.9, L40-L50.9, L52-L54.8, L56-L56.2, L56.4-L56.5, L57-L57.9, L59-L68.9, L70-L76.8, L80-L87.9, L90-L92.9, L94-L96, L98.5-L99.8, M04, M10-M12.0, M12.2-M29, M37-M39, M43.2-M49, M49.2-M64, M65.1-M71, M71.2-M72.4, M72.8-M73, M73.8-M79.9, M83-M86.2, M86.5-M86.9, M87.2-M87.9, M89.1-M89.4, M90-M99.9, N17-N17.9, N19-N19.9, N32.1-N32.2, N32.8-N33.8, N35-N35.9, N37-N37.8, N39.3-N39.8, N42-N43.4, N44.1-N44.8, N46-N48.9, N50-N53.9, N61-N64.9, N82-N82.9, N91-N91.5, N95, N95.1-N95.9, N97-N97.9, R02-R02.9, R03.1, R07.0, R08-R09, R09.3, R11-R12.0, R14-R19.6, R19.8-R23, R23.1-R30.9, R32-R50.1, R50.8-R57.9, R58.0-R72.9, R74-R78, R78.6-R94.8, R96-R99.9, U05, U07-U81, U89.9-U99, X40-X44.9, X46-X46.9, X49-X49.9, Y10-Y14.9, Y16-Y19.9, Z00-Z15.8, Z17-unsp.

Garbage Code (GBD Level 2)	A14.9, A29-A30.9, A45-A45.9, A47-A48, A48.8-A49, A49.3-A49.9, A61-A62, A72-A73, A76, A97, B08-B09, B11-B14, B28-B29, B31-B32.4, B34-B34.9, B61-B62, B68-B68.9, B73-B74.2, B76-B76.9, B78-B81.8, B84, B92-B94, B94.8-B94.9, B95.6-B97.3, B97.7-B99.9, D59, D59.4, D59.8-D59.9, F17-F17.9, G44.3, G91.3, G93.0, G93.3, I10-I10.9, I15-I15.9, I27, I27.8-I27.9, I50, I50.8-I50.9, I67.4, I70-I70.1, I70.9, I74-I75.8, J81, J81.1, J90-J90.0, J94-J94.1, J94.8-J94.9, K92.0-K92.2, N70-N71.9, N73-N74.0, N74.2-N74.8, R03-R03.0, R04-R06.9, R09.0-R09.2, R09.8-R10.9, R13-R13.9, R23.0, R58, S00-T98.3, W47-W48, W63, W71-W72, W76-W76.9, W82, W95-W97, W98, X07, X55-X56, X59-X59.9, Y20-Y34.9, Y86-Y87, Y87.2, Y89, Y89.9-Y99.9
Garbage Code (GBD Level 3)	A01, A31-A31.9, A42-A44.9, A49.2, A64-A64.0, A99-A99.0, B17, B17.1, B17.8-B17.9, B19-B19.0, B19.2-B19.9, B37-B46.9, B49-B49.9, B55, B55.1-B55.9, B58-B59.9, B89, B94.2, C14-C14.9, C22.9, C26-C29, C35-C36, C39-C39.9, C42, C46-C46.9, C55-C55.9, C57.9, C59, C63.9, C68, C68.9, C74-C74.9, C75.9-C80.9, C87, C97-D00.0, D01, D01.4-D02, D02.4-D02.9, D07, D07.3, D07.6-D09, D09.1, D09.7, D09.9-D10, D10.9, D13, D13.9-D14, D14.4, D17-D21.9, D28, D28.9-D29, D29.9-D30, D30.9, D36.0, D36.9-D37.0, D37.6-D38, D38.6-D39.0, D39.7, D39.9-D40, D40.9-D41, D41.9, D44, D44.9, D48, D48.7-D49.1, D49.5, D49.7-D49.9, D54, D75.9, D79-D85, D87-D88, D89.8-D99, E07.8-E08.9, E17-E19, E34.0, E34.9-E35.8, E37-E39, E47-E49., E62, E69, E87.7, E90-E998, F04-F06.1, F06.5-F07.0, F07.8-F08, F50, F50.8-F50.9, G09-G09.9, G15-G19, G21, G21.2, G21.4-G22.0, G27-G29, G33-G34, G38-G39., G42, G48-G49, G66-G69, G74-G79, G84-G88, G93.8-G94, G96-G96.9, G98-G98.9, I00.0, I03-I04., I14-I14., I16-I19, I29-I29.9, I44-I45.9, I49-I49.9, I51, I51.6-I59, I90-I94, I96-I96.9, I98.4-I98.8, I99-ID5.9, J02.9, J03.9, J04.3, J06, J06.9, J40-J40.9, J47-J59, J71-J79, J81.9, J83, J85.9, J87-J89, J90.9, J93.6, J97-J98.0, J98.4-J99.8, K21-K21.9, K22.7, K31.9-K34, K39, K47-K49, K53-K54, K63-K63.4, K63.8-K63.9, K69, K70.4-K70.9, K78-K79, K84, K87-K89, K92, K92.9-K93, K96-K99, L06-L07, L09, L15-L19, L31-L39, L69, L77-L79, N09, N13-N13.5, N13.7-N13.9, N24, N28.8-N28.9, N38, N39.9-N40.9, N54-N59, N66-N69, N78-N79, N84, N84.2-N86, N88-N90.9, N92-N94.9, N95.0, O08-O08.9, O17-O19, O27, O37-O39, O49-O59, O78-O79, O93-O95.9, P06, P16-P18, P30-P34.2, P40-P49, P62-P69, P73, P79, P82, P85-P89, P96.9-P99.9, Q08-Q10.3, Q19, Q29-Q29., Q36.0-Q36.9, Q46-Q49, Q88, Q89.9, Q94, Q99.9-R01.2, R07, R07.1-R07.9, R31-R31.9
Garbage Code (GBD Level 4)	B16.9, B64, B82-B82.9, B83.9, C69, C69.9, C91.1, C91.4-C91.5, C91.7-C91.9, C92.7-C92.9, C93.2, C93.5-C93.7, C93.9, E12-E14.9, G00, G00.9-G02.8, G03.9, I37.9, I42-I42.0, I42.9, I51.5, I64-I64.9, I67, I67.8-I68, I68.8-I69, I69.4-I69.9, J07-J08, J15.9, J17-J19.6, J22-J29, J64-J64.9, P23, P23.5-P23.9, P37.3-P37.4, R73-R73.9, V87-V87.1, V87.4-V88.1, V88.4-V89.9, V99-V99.0, X84-X84.9, Y09-Y09.9, Y85-Y85.9

Lista completa: https://ghdx.healthdata.org/record/ihme-data/gbd-2019-cause-icd-code-mappings

Quadro S2: Lista de causas-alvo utilizada na metodologia GBD-Brasil.

**Causas alvo**
pneumonia
anomalias congenitas
aspiracao pulmunar neonatal
dcnt_cardiovascular
dcnt_crônicas respiratórias
dcnt_diabetes
dcnt_neoplasms
infantil_neonatal_encefalopatia
infantil_neonatal_hemolitica
infantil_neonatal_outra
infantil_neonatal_prematuridade
infantil_neonatal_septicemia
infantil_subita
causas externas - quedas
causas externas - Homicídio
causas externas - outras causas externas de transporte
causas externas - outras
causas externas - trânsito
causas externas - Suicídio
Infecção respiratória inferior_pos_neonatal
materna_aborto_espontaneo
materna_aborto_induzido
materna_ectopica
materna_hemorragia
materna_hipertensiva
materna_indiretas
materna_materna_hiv
materna_outras
materna_septicemia
materna_tardia
materna_trabalho de parto
obstrução_intestinal
Todas as outras causas
outras causas-lri
outras_desnutricao_todas as idades
transmissíveis_chagas
transmissíveis_dengue
transmissíveis_desnutricao
transmissíveis_doenças_diarréicas
transmissíveis_encefatlite
transmissíveis_febre_amarela
transmissíveis_hepatite
transmissíveis_hiv_aids
transmissíveis_infec_urinaria
transmissíveis_leishmaniose
transmissíveis_malaria
transmissíveis_meningites
transmissíveis_esquistossomose
transmissíveis_sexualmente_transmissíveis
transmissíveis_tuberculose
transmissíveis_varicela
transmissíveis_zoonoticas

Quadro S3: algoritmo de distribuição das causas externas garbage X59 e Y34 pré-definido por reclassificação de causas dos óbitos investigados no Projeto 60 Cidades.

CG	target	age	sex	method	weight
_x59	Injuries - Falls	All ages	Both	investigation (pre defined wheights)	0,28788
_x59	Injuries - Homicide	All ages	Both	investigation (pre defined wheights)	0,03788
_x59	Injuries - Other transport injuries	All ages	Both	investigation (pre defined wheights)	0,02273
_x59	Injuries - Others	All ages	Both	investigation (pre defined wheights)	0,04545
_x59	Injuries - Road	All ages	Both	investigation (pre defined wheights)	0,48485
_x59	Injuries - Suicide	All ages	Both	investigation (pre defined wheights)	0,00758
_x59	outras causas naturais	All ages	Both	investigation (pre defined wheights)	0,11364
_x59	total				1,0
_y34	dcnt_cardiovascular	All ages	Both	investigation (pre defined wheights)	0,01132
_y34	dcnt_chronic respiratory	All ages	Both	investigation (pre defined wheights)	0,00204
_y34	dcnt_diabetes	All ages	Both	investigation (pre defined wheights)	0,00272
_y34	Injuries - Falls	All ages	Both	investigation (pre defined wheights)	0,17429
_y34	Injuries - Homicide	All ages	Both	investigation (pre defined wheights)	0,36849
_y34	Injuries - Other transport injuries	All ages	Both	investigation (pre defined wheights)	0,00249
_y34	Injuries - Others	All ages	Both	investigation (pre defined wheights)	0,03622
_y34	Injuries - Road	All ages	Both	investigation (pre defined wheights)	0,23631
_y34	Injuries - Suicide	All ages	Both	investigation (pre defined wheights)	0,05115
_y34	dcnt_neoplasms	All ages	Both	investigation (pre defined wheights)	0,00272
_y34	outras causas naturais	All ages	Both	investigation (pre defined wheights)	0,11227
_y34	total				1,0

Tabela S1: Correção das causas externas aplicando a redistribuição das CG de externas do Y34 e X59 com dados da investigação de óbitos (60 cidades) e a partir das causas definidas do SIM (pro rata), Brasil, 2019.

		correção			
	causas	algoritmo 60 cidades		proporcional de causas definidas (pro rata)	
		n	%	n	%
quedas	15.538	17.725	14,1	16.771	7,9
homicídios	44.033	47.644	8,2	49.050	11,4
trânsito	31.945	35.078	9,8	35.427	8,7
suicídios	13.520	14.025	3,7	13.547	7,3

Fonte: SIM/MS

Quadro S4: Ranking das taxas padronizadas de lesões de trânsito com dados diretos do SIM e o método GBD-Brasil, segundo estados, Brasil, 2019

Dados direto do SIM		GBD-Brasil	
1 Tocantins	27,5	1 Tocantins	32,5
2 Mato Grosso	25,6	2 Mato Grosso	27,9
3 Piauí	24,8	3 Piauí	27,1
4 Roraima	21,7	4 Rondônia	27,1
5 Rondônia	21,3	5 Roraima	24,1
6 Mato Grosso do Sul	20,5	6 Espírito Santo	24,1
7 Goiás	19,3	7 Maranhão	23,9
8 Paraná	18,9	8 Mato Grosso do Sul	22,4
9 Maranhão	17,9	9 Goiás	22,2
10 Santa Catarina	17,9	10 Alagoas	21,8
11 Paraíba	17,5	11 Paraná	21,3
12 Espírito Santo	17,5	12 Pernambuco	20,4
13 Alagoas	17,2	13 Paraíba	20,0
14 Sergipe	16,1	14 Santa Catarina	19,6
15 Pará	15,1	15 Sergipe	19,2
16 Bahia	14,2	16 Ceará	18,4
17 Pernambuco	14,1	17 Bahia	18,2
18 Ceará	13,9	18 Pará	16,6
19 Acre	13,6	19 Minas Gerais	16,3
20 Minas Gerais	12,9	20 Acre	14,7
21 Rio Grande do Sul	12,7	21 Rio de Janeiro	14,3
22 Rio Grande do Norte	11,6	22 Rio Grande do Sul	13,8
23 Amapá	10,5	23 Rio Grande do Norte	13,2
24 Amazonas	10,2	24 São Paulo	12,8
25 Distrito Federal	10,2	25 Distrito Federal	12,3
26 São Paulo	9,9	26 Amapá	12,0
27 Rio de Janeiro	7,4	27 Amazonas	10,9

Fonte: SIM/MS

Fonte: SIM/MS

Quadro S5: Ranking das taxas padronizadas de homicídios com dados diretos do SIM e o método GBD-Brasil, segundo estados, Brasil, 2019

Dados direto do SIM			GBD-Brasil		
1	Sergipe	37,7	1	Pernambuco	45,7
2	Roraima	36,4	2	Sergipe	45,2
3	Rio Grande do Norte	35,3	3	Bahia	43,9
4	Bahia	35,1	4	Roraima	42,8
5	Amazonas	34,9	5	Rio Grande do Norte	39,4
6	Amapá	34,8	6	Alagoas	38,3
7	Pará	34,0	7	Pará	38,1
8	Acre	33,4	8	Amazonas	38,1
9	Pernambuco	32,8	9	Amapá	37,3
10	Alagoas	30,3	10	Espírito Santo	35,8
11	Goiás	27,4	11	Acre	35,6
12	Tocantins	26,0	12	Goiás	32,5
13	Espírito Santo	24,6	13	Ceará	31,6
14	Ceará	23,6	14	Rondônia	31,2
15	Mato Grosso	23,4	15	Tocantins	30,8
16	Rondônia	22,5	16	Rio de Janeiro	30,6
17	Maranhão	22,0	17	Maranhão	27,2
18	Paraíba	21,7	18	Mato Grosso	26,8
19	Rio Grande do Sul	18,3	19	Paraíba	25,4
20	Rio de Janeiro	17,8	20	Rio Grande do Sul	20,3
21	Piauí	15,6	21	Paraná	19,2
22	Mato Grosso do Sul	15,4	22	Piauí	18,3
23	Paraná	15,3	23	Distrito Federal	18,2
24	Distrito Federal	14,1	24	Mato Grosso do Sul	17,8
25	Minas Gerais	12,6	25	Minas Gerais	17,7
26	Santa Catarina	9,5	26	Santa Catarina	11,0
27	São Paulo	6,3	27	São Paulo	10,2

Fonte: SIM/MS

Quadro S6: Ranking das taxas padronizadas de quedas com dados diretos do SIM e o método GBD-Brasil, segundo estados, Brasil, 2019

Dados direto do SIM		GBD-Brasil	
1 Roraima	15,1	1 Roraima	17,8
2 Goiás	13,3	2 Goiás	15,3
3 Paraná	12,0	3 Espírito Santo	15,2
4 Espírito Santo	11,5	4 Paraná	13,4
5 Distrito Federal	10,6	5 Tocantins	12,4
6 Tocantins	9,7	6 Pernambuco	12,3
7 Sergipe	9,4	7 Sergipe	12,2
8 Alagoas	9,1	8 Distrito Federal	12,1
9 Paraíba	9,0	9 Alagoas	11,7
10 Santa Catarina	8,9	10 Paraíba	10,9
11 Mato Grosso	8,4	11 Santa Catarina	10,0
12 Pernambuco	8,2	12 Minas Gerais	9,7
13 Rio Grande do Sul	7,8	13 Rondônia	9,6
14 Minas Gerais	7,5	14 Rio de Janeiro	9,4
15 Rondônia	7,1	15 Mato Grosso	9,4
16 São Paulo	6,5	16 São Paulo	9,0
17 Mato Grosso do Sul	6,2	17 Ceará	8,9
18 Amapá	6,0	18 Rio Grande do Sul	8,9
19 Pará	5,9	19 Maranhão	8,1
20 Amazonas	5,5	20 Mato Grosso do Sul	8,0
21 Ceará	5,3	21 Bahia	7,8
22 Bahia	4,6	22 Pará	7,7
23 Maranhão	4,6	23 Amapá	7,4
24 Piauí	4,6	24 Amazonas	6,7
25 Rio de Janeiro	4,1	25 Piauí	6,5
26 Rio Grande do Norte	3,9	26 Rio Grande do Norte	5,8

Fonte: SIM/MS

Quadro S7: Ranking das taxas padronizadas de suicídios com dados diretos do SIM e o método GBD-Brasil, segundo estados, Brasil, 2019

Dados direto do SIM		GBD-Brasil	
1 Rio Grande do Sul	10,5	1 Rio Grande do Sul	11,3
2 Santa Catarina	9,7	2 Santa Catarina	10,9
3 Piauí	9,3	3 Mato Grosso do Sul	10,1
4 Mato Grosso do Sul	8,9	4 Piauí	10,1
5 Tocantins	8,0	5 Tocantins	9,7
6 Acre	7,8	6 Rondônia	9,5
7 Roraima	7,5	7 Paraná	8,7
8 Goiás	7,5	8 Minas Gerais	8,6
9 Paraná	7,4	9 Goiás	8,6
10 Rondônia	7,4	10 Roraima	8,5
11 Minas Gerais	7,1	11 Acre	8,4
12 Amapá	6,7	12 Ceará	8,0
13 Mato Grosso	6,5	13 Espírito Santo	7,7
14 Ceará	6,3	14 Mato Grosso	7,2
15 Distrito Federal	5,9	15 Amapá	7,2
16 Amazonas	5,7	16 Distrito Federal	7,1
17 Paraíba	5,6	17 Paraíba	6,5
18 Espírito Santo	5,6	18 São Paulo	6,1
19 Rio Grande do Norte	5,3	19 Rio Grande do Norte	6,1
20 Sergipe	4,7	20 Amazonas	6,1
21 São Paulo	4,6	21 Pernambuco	5,9
22 Maranhão	4,5	22 Maranhão	5,8
23 Pernambuco	4,2	23 Sergipe	5,8
24 Bahia	3,9	24 Bahia	5,3
25 Pará	3,8	25 Rio de Janeiro	4,8
26 Alagoas	3,6	26 Alagoas	4,5
27 Rio de Janeiro	2,9	27 Pará	4,4

Fonte: SIM/MS
